# Circ_0072083 interference enhances growth-inhibiting effects of cisplatin in non-small-cell lung cancer cells via miR-545-3p/CBLL1 axis

**DOI:** 10.1186/s12935-020-1162-x

**Published:** 2020-03-12

**Authors:** Huiping Li, Fangfang Liu, Wenjing Qin

**Affiliations:** Department of Respiratory Medicine, First Affiliated Hospital of Henan University, No. 357, Ximen Street, Longting District, Kaifeng, 475000 Henan China

**Keywords:** NSCLC, circ_0072083, miR-545-3p, CBLL1, DDP

## Abstract

**Background:**

Non-small-cell lung cancer (NSCLC) is one of the common cancers in the world. Circular RNA 0072083 (circ_0072083, circZFR) has been reported to be associated with the progression of NSCLC. In this study, we intended to explore the role and the potential mechanism of circ_0072083 in NSCLC.

**Methods:**

Quantitative real time polymerase chain reaction (qRT-PCR) was performed to detect the expression of circ_0072083, its matching linear RNA (zinc finger RNA binding protein (ZFR)) and microRNA-545-3p (miR-545-3p) in NSCLC cells. The ability of colony formation in NSCLC cells was detected by colony formation assay. The apoptosis and cell cycle were measured by flow cytometry. The metastasis was determined by transwell migration and invasion assays. The protein expression of E-cadherin, N-cadherin, Vimentin and Cbl proto-oncogene like 1 (CBLL1) was examined by western blot assay. The interaction between miR-545-3p and circ_0072083 or CBLL1 was predicted by starBase or Targetscan software. Dual-luciferase reporter assay and RNA immunoprecipitation (RIP) assay were applied to validate these interactions. Nude mice bearing tumors were used to confirm the role of circ_0072083 and cisplatin (DDP) in vivo.

**Results:**

The level of circ_0072083 was higher in NSCLC tissues and cells relative to that in adjacent non-tumor tissues and normal lung cells. The transfection of si-circ_0072083 inhibited colony formation, cell cycle and metastasis while promoted the apoptosis of NSCLC cells stimulated by DDP. MiR-545-3p was a direct functional target of circ_0072083 in NSCLC cells. CBLL1 could bind to miR-545-3p in NSCLC cells. Circ_0072083 promoted the progression of NSCLC induced by DDP through sponging miR-545-3p and enhancing the enrichment of CBLL1 in vivo and in vitro.

**Conclusion:**

Circ_0072083 depletion contributed to DDP-triggered inhibition of NSCLC tumor through miR-545-3p/CBLL1 axis.

## Background

Lung cancer is the leading cause of the cancer-associated mortality globally [1, 2]. There were 2093,876 new cases and 1,761,007 deaths of lung cancer globally in 2018, accounting for 11.6% and 18.4% of all cancers, respectively [2]. Lung cancer contains small-cell lung cancer (SCLC) and non-small-cell lung cancer (NSCLC). Among the two types of lung cancer, NSCLC accounts for 85% [3]. The therapeutic methods for NSCLC patients include surgery resection, chemotherapy, radiotherapy, targeted therapy and immunotherapy [4]. In the case of chemotherapy, the chemoresistance of NSCLC patients remains a big obstacle for the curing the NSCLC. Therefore, uncovering the resistance mechanism of NSCLC cells is urgent to avoid the metastasis or neoplasm recurrence.

Circular RNAs (circRNAs) are a class of non-coding RNAs, and they possess the closed loop structure [5]. CircRNAs are crucial modulators in cancers [6–8]. Circ_0072083 (circZFR) could regulate the progression of multiple cancers through acting as microRNAs (miRNAs) sponges. Wei et al. proved that circ_0072083 promoted the development of papillary thyroid cancer through miR-1261/C8orf4 axis [9]. Tan et al. reported that circ_0072083 contributed to the progression of hepatocellular cancer via miR-3619-5p/CTNNB1 signaling [10]. Wang et al. demonstrated that circ_0072083 accelerated the proliferation and metastasis of human renal carcinoma via sponging miR-206 [11]. Zhang et al. found that circ_0072083 acted as an oncogene in NSCLC via miR-101-3p/CUL4B axis [12]. Nevertheless, the working network of circ_0072083 in NSCLC remains not fully elucidated.

MiRNAs have emerged as pivotal regulators in cancers. Muhammad et al. found that miR-203 served as an oncogene in ER-positive breast cancer, and it accelerated the growth and stemness of ER-positive breast cancer via SOCS3 [13]. Fan et al. demonstrated that miR-125a impeded the proliferation and motility of cervical cancer cells through suppressing STAT3 [14]. Du et al. reported that miR-33a inhibited the proliferation of NSCLC cells through METTL3 [15]. Wang et al. proved that miR-204 repressed the motility of NSCLC cells via JAK2 [16]. Besides, miR-545 has been reported to hamper the progression of NSCLC via ZEB2 [17]. However, the interaction between circ_0072083 and miR-545-3p in NSCLC has not been reported.

Cbl proto-oncogene like 1 (CBLL1) is an E3 ubiquitin ligase with RING-finger domain. Accumulating articles have reported the role of CBLL1 in NSCLC. Hui et al. found that the expression of CBLL1 was higher in NSCLC, and it facilitated the growth and motility of NSCLC cells [18]. Qui et al. reported that XIST accelerated the proliferation and metastasis of NSCLC cells via acting as a miR-212-3p sponge and up-regulating CBLL1 [19]. In the present study, we investigated whether miR-545-3p exerted its function through CBLL1 in NSCLC.

We measured the abundance of circ_0072083, miR-545-3p and CBLL1 in NSCLC tissues and cells. Moreover, we illustrated the mechanism by which circ_0072083 contributed to the chemoresistance of NSCLC cells to cisplatin (DDP).

## Materials and methods

### Clinical samples

The NSCLC tumor tissues and adjacent non-tumor tissues were acquired from a total of 31 NSCLC patients in First Affiliated Hospital of Henan University. Written informed consents were obtained from all subjects in this study. The utilization of human material was approved by the Institute Research Ethics Committee of First Affiliated Hospital of Henan University.

### Cell culture and DDP treatment

Human NSCLC cell lines (H522 and A549) and normal human lung epithelial cell line BEAS-2B were purchased from BeNa Culture Collection (Beijing, China) and cultivated in Dulbecco’s Modified Eagle’s Medium (DMEM, Gibco, Carlsbad, CA, USA) added with 10% heat-inactivated fetal bovine serum (FBS, Gibco), 100 units/mL penicillin and 100 μg/mL streptomycin under 37 °C humidified incubator with 5% CO_2_. 30 µg/mL DDP was used to stimulate NSCLC cells for 48 h.

### Quantitative real-time polymerase chain reaction (qRT-PCR)

qRT-PCR was conducted to detect the enrichment of relevant RNAs. RNA sample was isolated with TRIzol (Beyotime, Shanghai, China). Reverse transcription was conducted using High-Capacity cDNA Reverse Transcription Kit (Applied Biosystems, Foster City, CA, USA). Hieff Unicon aqMan multiplex qPCR master mix (YEASEN, Shanghai, China) was used to conduct qRT-PCR reaction. Glyceraldehyde-3-phosphate dehydrogenase (GAPDH) or U6 was acted as the internal controls. The abundance of RNA was calculated using the formula of 2^−ΔΔCt^. The primer sequences were listed as below: circ_0072083, Forward primer (FP), 5′-AACCACCACAGATTCACTAT-3′, Reverse primer (RP), 5′-AACCACCACAGATTCACTAT-3′; zinc finger RNA binding protein (ZFR; circ_0072083 matching linear RNA), FP, 5′-GCCCAGCCGGCTGGGGGAAG-3′, RP, 5′-GCAGCTACTGGAGCCTGATG-3′; miR-545-3p, FP, 5′-TGGCTCAGTTCAGCAGGAAC-3′, RP, universal reverse primer; GAPDH, FP, 5′-ATAGCACAGCCTGGATAGCAACGTAC-3′, RP, 5′-CACCTTCTACAATGAGCTGCGTGTG-3′; U6, FP, 5′-CGCTTCGGCAGCACATATAC-3′, RP, 5′-TTCACGAATTTGCGTGTCAT-3′.

### RNase R treatment

To confirm the loop structure of circ_0072083, RNase R was used in this study. 40 U RNase R was incubated with 10 μg RNA sample for 15 min, and the abundance of circ_0072083 and ZFR was determined through performing qRT-PCR assay.

### Cell transfection

NSCLC cells were plated in 6-well plates and transfected with the following RNA or plasmid with lipofectamine 3000 (Invitrogen, Carlsbad, CA, USA). Circ_0072083 specific small interfering RNA (si-circ_0072083), siRNA negative control (si-NC), circ_0072083 specific short hairpin RNA (Sh-circ_0072083) and its control, miR-545-3p mimic (miR-545-3p), miR-NC, miR-545-3p inhibitor (anti-miR-545-3p) and its matching control (anti-miR-NC), CBLL1 plasmid (CBLL1) and its matching empty vector (vector) were obtained from Genepharma (Shanghai, China).

### Colony formation assay

A549 or H522 cells were transferred into 6-well plates at a concentration of 150 cells/well. The cells were cultured for 2 weeks until the cell colonies were visible, and the culture medium was replaced with fresh medium every 3 days. The colonies were immobilized and then stained using crystal violet.

### The apoptosis detection by flow cytometry

Annexin-V-fluorescein isothiocyanate (FITC) Apoptosis Detection Kit (R&D Systems, Abingdon, UK) was utilized to detect the apoptosis of NSCLC cells. Briefly, NSCLC cells were collected and then re-suspended in 100 μL binding buffer. Annexin-V-FITC and propidium iodide (PI) were incubated with NSCLC cells in the dark for 15 min. The apoptosis of NSCLC cells was analyzed on FC-500 flow cytometer (Beckman Coulter, Pasadena, CA, USA).

### Cell cycle detection by flow cytometry

NSCLC cells were immobilized with 70% ethanol at − 20 °C overnight. Subsequently, the cells were incubated with RNase for 30 min at 37 °C to remove the RNA. PI was added to the cells to stain the DNA at 4 °C for 1 h. FC-500 flow cytometer (Beckman Coulter) was used to detect the cell cycle.

### Transwell migration and invasion assays

Transwell chambers were purchased from Millipore (Billerica, MA, USA). To detect the migration of NSCLC cells, A549 and H522 cells in serum-free medium were transferred into the upper chambers with the non-coated membrane. To detect the invasion of NSCLC cells, the upper chambers were pre-coated with 100 μL Matrigel (BD Biosciences, San Jose, CA, USA). 10% FBS was added into the lower chambers to act as chemotactic factor. The migrated or invaded cells in the lower face of the membrane were immobilized and then dyed with 1% crystal violet. The cell numbers in three random fields were counted using an optical microscope.

### Western blot assay

H522 and A549 cells were harvested and lysed using cell lysis buffer (Beyotime). The concentration of protein samples was determined using bicinchoninic acid (BCA). A total of 30 μg protein sample was separated using 10% sodium dodecyl sulfate polyacrylamide gel electrophoresis (SDS-PAGE) and transferred into polyvinylidene difluoride (PVDF) membranes (Millipore). The membranes were blocked using 5% skim milk for 1 h, and then the membranes were probed with the following antibodies at 4 °C overnight: anti-E-cadherin (ab40772, Abcam, Cambridge, MA, USA), anti-N-cadherin (ab18203, Abcam), anti-Vimentin (ab92547, Abcam), anti-CBLL1 (ab50993, Abcam), anti-Bcl-2 (ab185002, Abcam), anti-Bax (ab32503, Abcam) and anti-GAPDH (ab181602, Abcam). The secondary antibody was then incubated with the membranes for 2 h at room temperature. The protein expression was measured by the enhanced chemiluminescence (ECL) system (Millipore).

### Lactate dehydrogenase (LDH) cytotoxicity assay

NSCLC cells were plated into 96-well plates, and LDH releasing reagent (20 µL; LDH cytotoxicity assay kit; Beyotime) was added to the wells of 96-well plates. NSCLC cells were incubated with LDH releasing reagent for 1 h followed by centrifugation for 5 min at 400×*g*. The cell supernatant (120 µL) of each well was transferred to new 96-well plates, and the optical density was detected at 490 nm.

### Dual-luciferase reporter assay

The miR-545-3p binding sites in circ_0072083 (wile type or mutant type) or the 3′ untranslated region (3′ UTR) of CBLL1 (wile type or mutant type) were cloned to pmirGLO vector (Promega, Madison, WI, USA), termed as circ_0072083 WT or circ_0072083 MUT and CBLL1 3′ UTR WT or CBLL1 3′ UTR MUT. The luciferase activity was analyzed through the dual-Luciferase Reporter Assay System (Promega).

### RNA immunoprecipitation (RIP) assay

Magna RIP RNA-Binding Protein Immunoprecipitation Kit (Millipore) was used in RIP assay to verify the combination between circ_0072083 and miR-545-3p. The NSCLC cells were incubated with protein-A/G Sepharose beads (Bio-Rad, Hercules, CA, USA) pre-coated with Argonaute-2 antibody (Anti-Ago2) or Immunoglobulin G antibody (Anti-IgG). The complexes were pulled-down by the protein-A/G Sepharose beads, and qRT-PCR was carried out to do the following detection.

### Animal study

Nude mice purchased from Laboratory Animal Center of the Academy of Military Medical Sciences (Beijing, China) were inoculated with H522 cells stably expressing Sh-circ_0072083. 3 mg/kg DDP or phosphate buffered saline (PBS) was intravenously injected into the mice every 5 days after inoculation for 5 days. The size of tumors was measured every 5 days. The nude mice were sacrificed and the tumors were resected after inoculation for 30 days. And then the tumors were weighed, and the enrichment of circ_0072083, miR-545-3p and CBLL1 was detected by qRT-PCR and Western blot. The animal study was performed with the permission of the Institutional Animal Care and Use Committee at First Affiliated Hospital of Henan University.

### Statistical analysis

Data were presented as mean ± standard deviation (SD). Student’s *t* test or one-way analysis of variance (ANOVA) followed by Tukey’s test was used to analyze the differences as appropriate. *P *< 0.05 was considered statistically significant.

## Results

### Circ_0072083 is aberrantly up-regulated in NSCLC specimens and cells

**Fig. 1:**
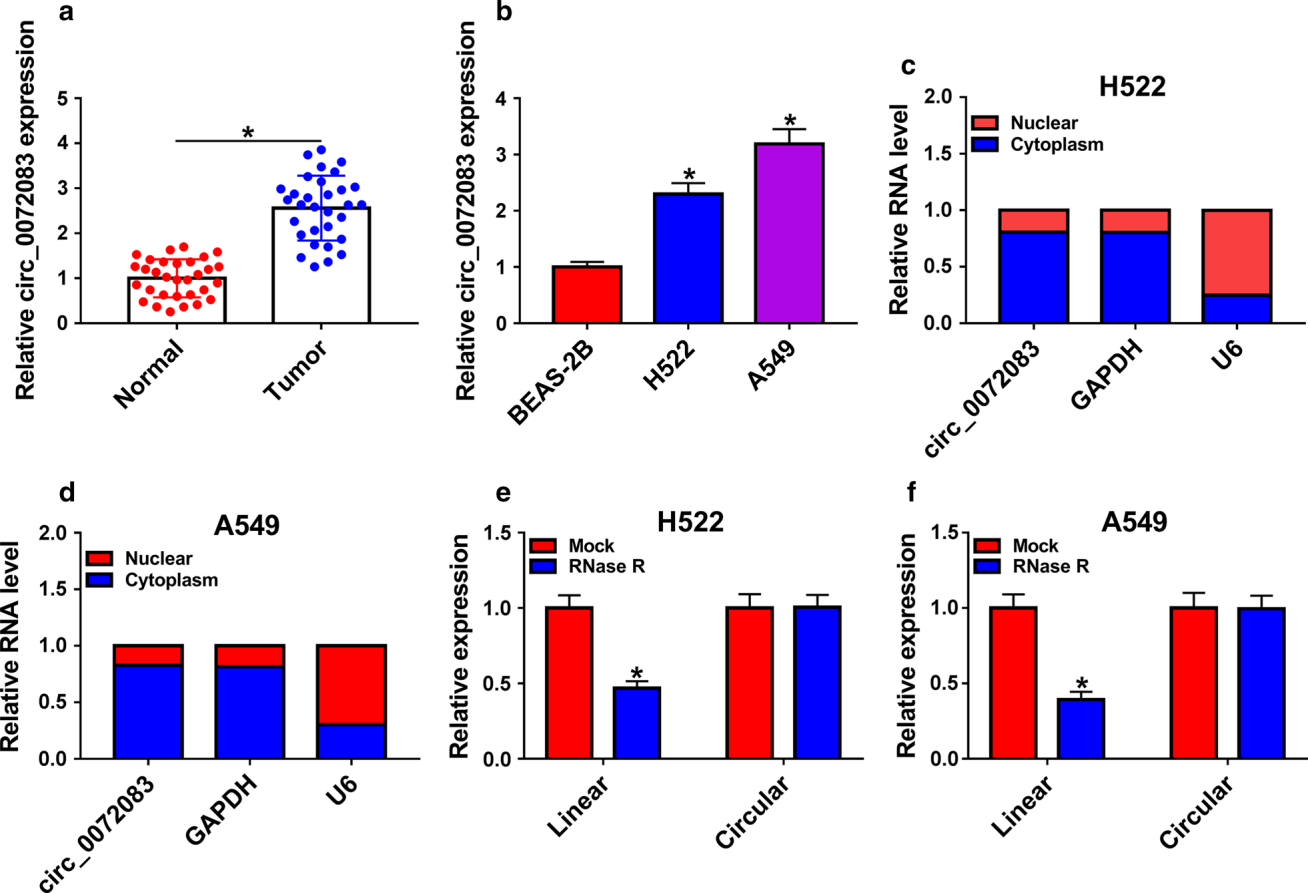
Circ_0072083 is aberrantly up-regulated in NSCLC specimens and cells. **a** Expression level of circ_0072083 was detected in NSCLC samples and adjacent normal tissues by qRT-PCR. **b** qRT-PCR was performed to measure the expression of circ_0072083 in normal human lung epithelial cell line BEAS-2B and NSCLC cell lines (H522 and A549). **c**, **d** The distribution of circ_0072083 in the nuclear or cytoplasm fraction of NSCLC cells was determined by qRT-PCR. **e, f** The stability of circ_0072083 was assessed in the control group and RNase R group of A549 and H522 cells by qRT-PCR. *P < 0.05

To determine the role of circ_0072083 in NSCLC, the expression of circ_0072083 in NSCLC tissues and cell lines and their corresponding normal controls was examined. As depicted in Fig. 1a, b, abnormal up-regulation of circ_0072083 was observed in NSCLC tissues and cells relative to normal tissues and normal human lung epithelial cells BEAS-2B. We also explored the subcellular distribution of circ_0072083. Circ_0072083 mainly distributed in the cytoplasmic fraction of H522 and A549 cells (Fig. 1c, d). The stability of circ_0072083 was measured in NSCLC cells treated with RNase R. Compared with matching linear messenger RNA (mRNA; ZFR), circ_0072083 was more stable owing to its closed loop structure (Fig. 1e, f).

### Circ_0072083 knockdown decreases the DDP resistance of NSCLC cells

**Fig. 2:**
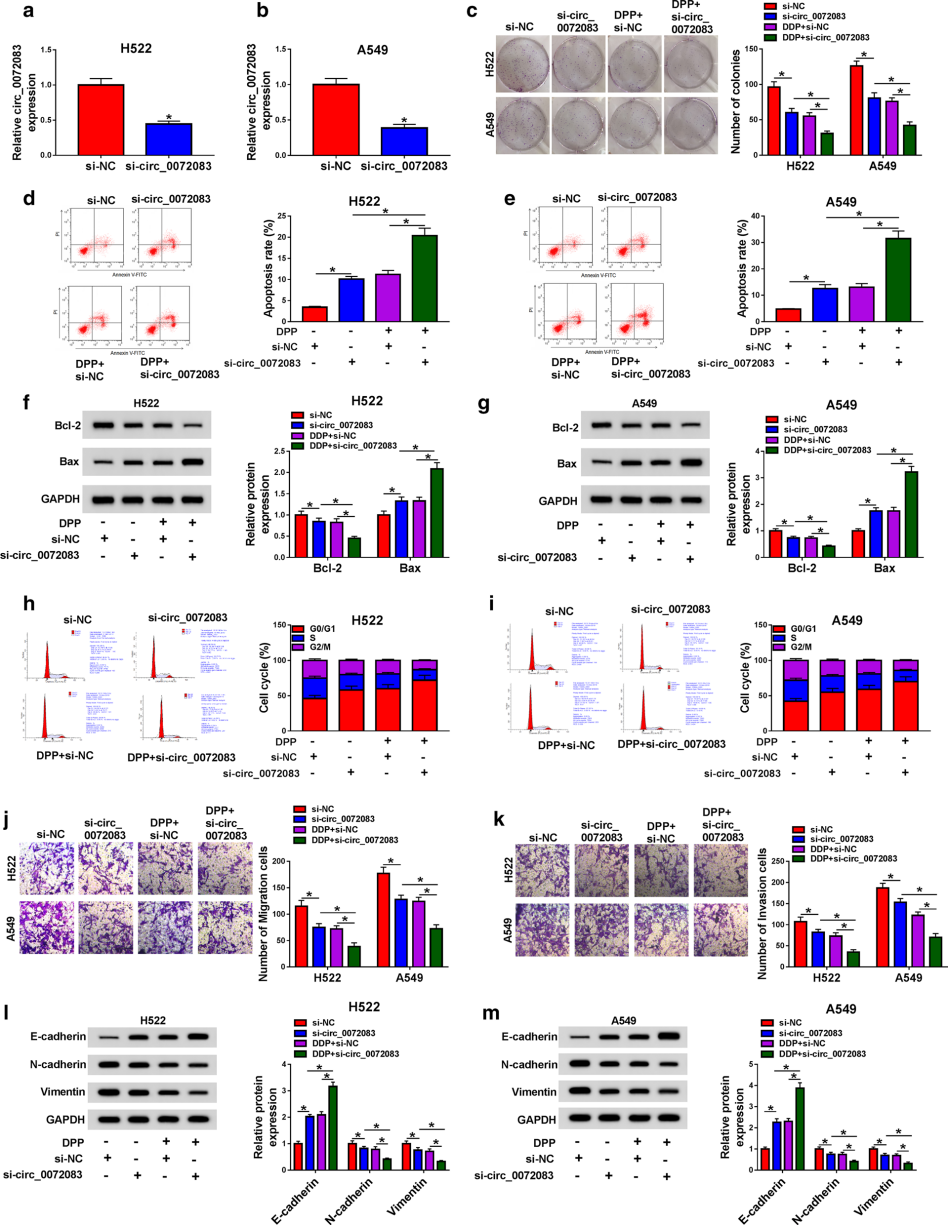
Circ_0072083 knockdown decreases the DDP resistance of NSCLC cells. **a**, **b** The level of circ_0072083 was detected in H522 and A549 cells transfected with si-NC or si-circ_0072083 by qRT-PCR. **c–m** H522 and A549 cells were treated with si-NC, si-circ_0072083, DDP + si-NC or DDP + si-circ_0072083. **c** The colony formation ability was detected in NSCLC cells through colony formation assay. **d, e** The apoptosis rate of NSCLC cells was evaluated by flow cytometry. **f**, **g** Western blot assay was carried out to detect the apoptosis-related markers in NSCLC cells. **h**, **i** Cell cycle of NSCLC cells was analyzed by flow cytometry. **j**, **k** The motility of NSCLC cells was detected through conducting transwell migration and invasion assays. **l, m** Western blot assay was performed to detect the protein expression of E-cadherin, N-cadherin and Vimentin in NSCLC cells, and GAPDH served as the internal reference in this study. *P < 0.05

To further clarify the function of circ_0072083 in NSCLC, circ_0072083 was silenced in H522 and A549 cells through transfecting si-circ_0072083 into the two cells. There was a significant decrease in the level of circ_0072083 in si-circ_0072083 transfected group (Fig. 2a, b). Next, we examined the effects of circ_0072083 knockdown on the colony formation, apoptosis, cell cycle and metastasis of NSCLC cells exposed to DDP. The capacity of colony formation in NSCLC cells was inhibited with the depletion of circ_0072083, and the capacity was further decreased with the addition of DDP (Fig. 2c). The apoptosis rate of NSCLC cells exhibited a reverse phenomenon to the colony formation (Fig. 2d, e). The changes in the expression of pro-apoptotic protein Bax and anti-apoptotic protein Bcl-2 revealed that circ_0072083 depletion accelerated the apoptosis, and the co-treatment of si-circ_0072083 and DDP further exacerbated the apoptosis of NSCLC cells (Fig. 2f, g). We also investigated the influence of circ_0072083 silencing on the cell cycle of NSCLC cells according to the cell cycle stage distribution (G0/G1, S, G2/M). As indicated in Fig. 2h, i, there was an up-regulation of the cell percentage at G0/G1 phase, suggesting that circ_0072083 depletion arrested cell cycle at G0/G1 phase. Moreover, the results of transwell migration and invasion assays showed that DDP further aggravated circ_0072083 silencing-mediated inhibition of metastasis in NSCLC cells (Fig. 2j, k). Epithelial-mesenchymal transition (EMT) markers, including E-cadherin, N-cadherin and Vimentin, were detected in H522 and A549 cells treated with si-NC, si-circ_0072083, DDP + si-NC or DDP + si-circ_0072083. The expression of N-cadherin and Vimentin was decreased with the intervention of circ_0072083, and the introduction of DDP exacerbated the inhibitory effect caused by circ_0072083 inhibition (Fig. 2l, m). The abundance of E-cadherin revealed an opposite trend to N-cadherin or Vimentin, suggesting that DDP promoted the suppressive influence of circ_0072083 depletion on the metastasis of NSCLC cells. Besides, the results of LDH cytotoxicity assay suggested that DDP promoted si-circ_0072083-mediated necrosis of NSCLC cells (Additional file 1: Figure S1). The knockdown of circ_0072083 had no significant effects on the colony formation and apoptosis of normal human lung epithelial cells BEAS-2B (Additional file 2: Figure S2).

### Circ_0072083 could modulate the expression of miR-545-3p through directly binding to miR-545-3p

**Fig. 3:**
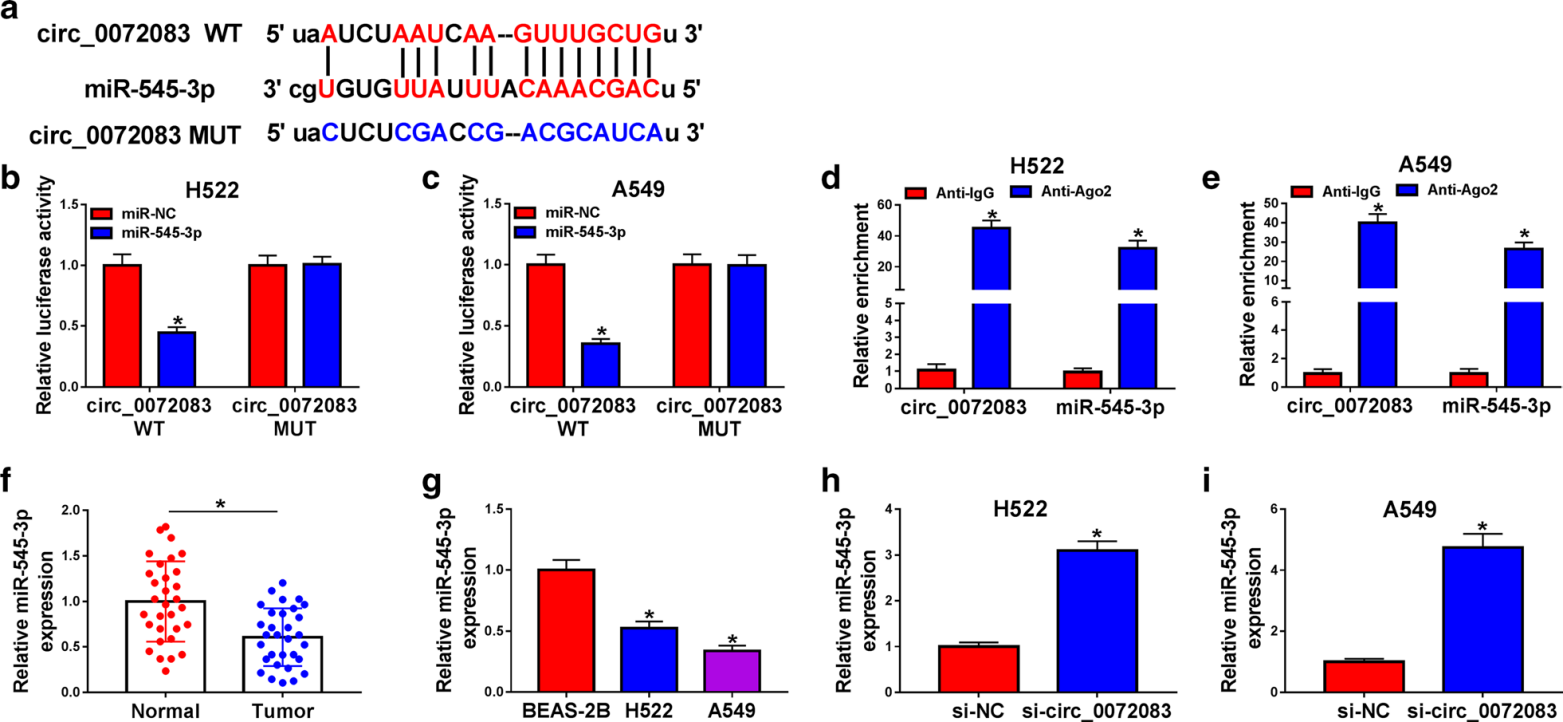
Circ_0072083 could modulate the expression of miR-545-3p through directly binding to miR-545-3p. **a** The complementary sites marked in red between miR-545-3p and circ_0072083 were predicted by starBase database. **b**, **c** Dual-luciferase reporter assay was carried out to verify the target relationship between miR-545-3p and circ_0072083. **d, e** RIP assay was performed to validate the combination between miR-545-3p and circ_0072083. **f** The expression of miR-545-3p in NSCLC tissues and corresponding normal tissues was determined by qRT-PCR. **g** The level of miR-545-3p in a panel of three lung cell lines, including normal human lung epithelial cell line BEAS-2B and two NSCLC cell lines (H522 and A549) was examined by qRT-PCR. **h, i** The relative expression of miR-545-3p in si-NC or si-circ_0072083 transfected NSCLC cells was examined by qRT-PCR. *P < 0.05

According to the prediction from starBase online software, there existed binding sites between circ_0072083 and miR-545-3p (Fig. 3a). The results of dual-luciferase reporter assay revealed that the co-transfection of miR-545-3p and circ_0072083 WT remarkably declined the luciferase activity, while it remained unchanged in miR-545-3p and circ_0072083 MUT group (Fig. 3b, c). RIP experiment suggested that both circ_0072083 and miR-545-3p existed in RNA induced silencing complex (RISC) (Fig. 3d, e).

Next, we found that miR-545-3p was down-regulated in NSCLC tissues compared with that in adjacent normal tissues (Fig. 3f). Meanwhile, the low expression of miR-545-3p was also observed in the two NSCLC cell lines in comparison with that in BEAS-2B cells (Fig. 3g). To explore the role of circ_0072083 on the expression of miR-545-3p in NSCLC cells, circ_0072083 siRNA was transfected into NSCLC cells. As shown in Fig. 3h, i, miR-545-3p was up-regulated with the depletion of circ_0072083 in NSCLC cells.

### MiR-545-3p depletion overturns the effects of circ_0072083 knockdown on NSCLC cells treated with DDP

**Fig. 4:**
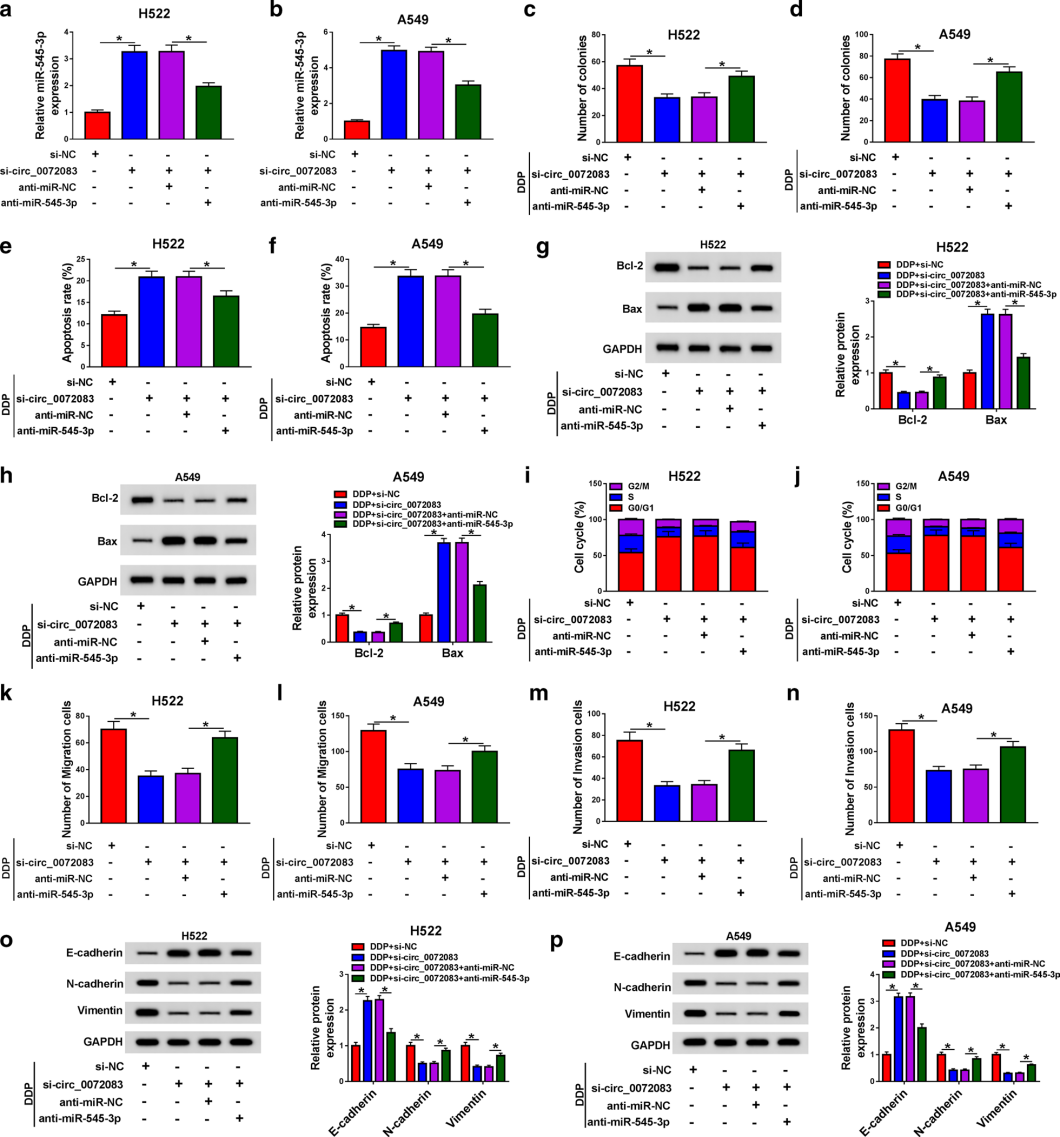
MiR-545-3p depletion overturns the effects of circ_0072083 knockdown in NSCLC cells treated with DDP. We transfected si-NC, si-circ_0072083, si-circ_0072083 + anti-miR-NC or si-circ_0072083 + anti-miR-545-3p into H522 and A549 cells. **a, b** The expression of miR-545-3p was detected in NSCLC cells by qRT-PCR. **c, d** The number of colonies was counted through colony formation assay in NSCLC cells treated with DDP. **e, f** The apoptosis of NSCLC cells treated with DDP was assessed by flow cytometry. **g, h** The expression of Bcl-2 and Bax was determined in NSCLC cells by western blot assay. **i, j** Flow cytometry was used to analyze the cell cycle of NSCLC cells treated with DDP. **k, l** Transwell migration assay was conducted to detect the migration of NSCLC cells treated with DDP. **m, n** The capacity of invasion in NSCLC cells treated with DDP was determined by transwell invasion assay. **o, p** Motility-associated proteins, such as E-cadherin, N-cadherin and Vimentin, were detected in NSCLC cells treated with DDP by western blot assay. *P < 0.05

To illustrate whether circ_0072083 exerted its role through sponging miR-545-3p in NSCLC cells, we conducted rescue experiments. As exhibited in Fig. 4a, b, circ_0072083 knockdown increased the abundance of miR-545-3p in NSCLC cells, and this promoting impact was reversed by the addition of anti-miR-545-3p. Further investigation showed that the inhibitory effects on the colony formation, cell cycle and metastasis along with the promoting influence on the apoptosis of DDP-induced NSCLC cells caused by circ_0072083 interference were overturned by the co-transfection of si-circ_0072083 + anti-miR-545-3p (Fig. 4c–n). Besides, western blot assay revealed that the addition of anti-miR-545-3p recovered the ability of metastasis, which was suppressed by the intervention of circ_0072083 in DDP-stimulated NSCLC cells (Fig. 4o, p). Furthermore, the addition of anti-miR-545-3p recovered the viability of NSCLC cells stimulated by DDP which was restrained by the interference of circ_0072083 (Additional file 1: Figure S1b).

### CBLL1 binds to miR-545-3p in NSCLC cells

**Fig. 5:**
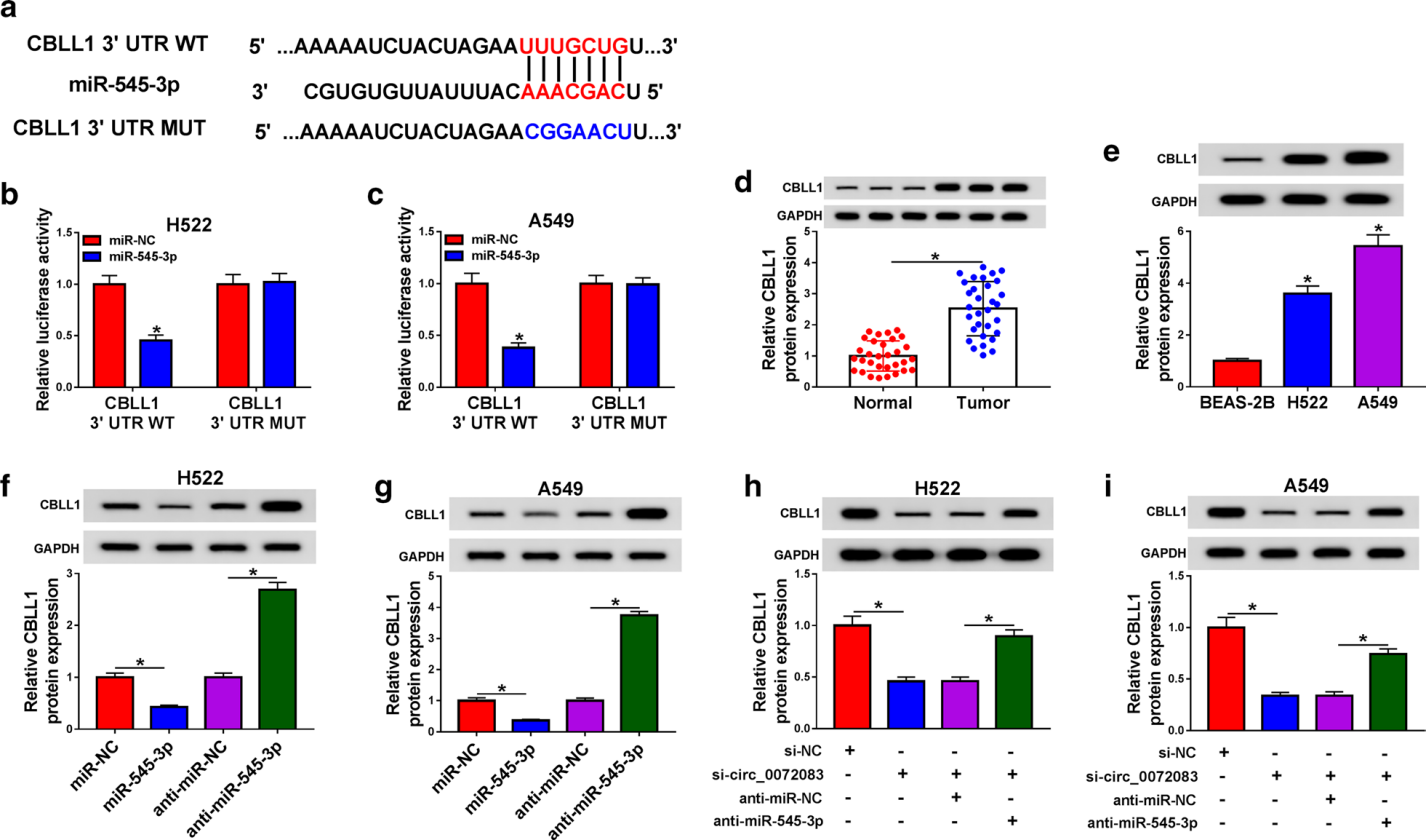
CBLL1 binds to miR-545-3p in NSCLC cells. **a** The 3′ UTR of CBLL1 possessed the complementary bases with miR-545-3p using Targetscan software. **b, c** Luciferase activity was determined in H522 and A549 cells co-transfected with miR-NC or miR-545-3p and CBLL1 3′ UTR WT or CBLL1 3′ UTR MUT reporter plasmid. **d** The protein expression of CBLL1 was examined in NSCLC samples and matching non-tumor tissues by western blot assay. **e** The level of CBLL1 was measured in BEAS-2B, H522 and A549 cells by western blot assay. **f, g** H522 and A549 cells were transfected with miR-NC, miR-545-3p, anti-miR-NC or anti-miR-545-3p, and Western blot was performed to detect the level of CBLL1 in the above NSCLC cells. **h, i** The expression of CBLL1 was detected in NSCLC cells transfected with si-NC, si-circ_0072083, si-circ_0072083 + anti-miR-NC or si-circ_0072083 + anti-miR-545-3p by western blot assay. *P < 0.05

Targetscan software predicted that CBLL1 might be a target of miR-545-3p (Fig. 5a). To validate this prediction, dual-luciferase reporter assay was carried out. As depicted in Fig. 5b, c, miR-545-3p transfection declined the luciferase activity in CBLL1 3′ UTR WT group other than CBLL1 3′ UTR MUT group.

There was a higher abundance of CBLL1 in NSCLC tissues relative to matching non-tumor tissues (Fig. 5d). Also, the expression of CBLL1 was higher in NSCLC cells than that in BEAS-2B cells (Fig. 5e). MiR-545-3p mimic and anti-miR-545-3p were transfected into NSCLC cells, and Western blot was performed to detect the protein expression of CBLL1 in the two NSCLC cells. MiR-545-3p accumulation caused a decrease in the expression of CBLL1 in NSCLC cells, while the transfection of anti-miR-545-3p up-regulated the expression of CBLL1 (Fig. 5f, g). As mentioned above, miR-545-3p was a direct target of circ_0072083 in NSCLC cells, and we wondered whether CBLL1 was modulated by circ_007208/miR-545-3p axis. The enrichment of CBLL1 was declined by the transfection of si-circ_0072083 in NSCLC cells, and the level of CBLL1 was regained with the introduction of anti-miR-545-3p (Fig. 5h, i).

### MiR-545-3p inhibits the malignant potential of NSCLC cells stimulated with DDP via inhibiting CBLL1

**Fig. 6:**
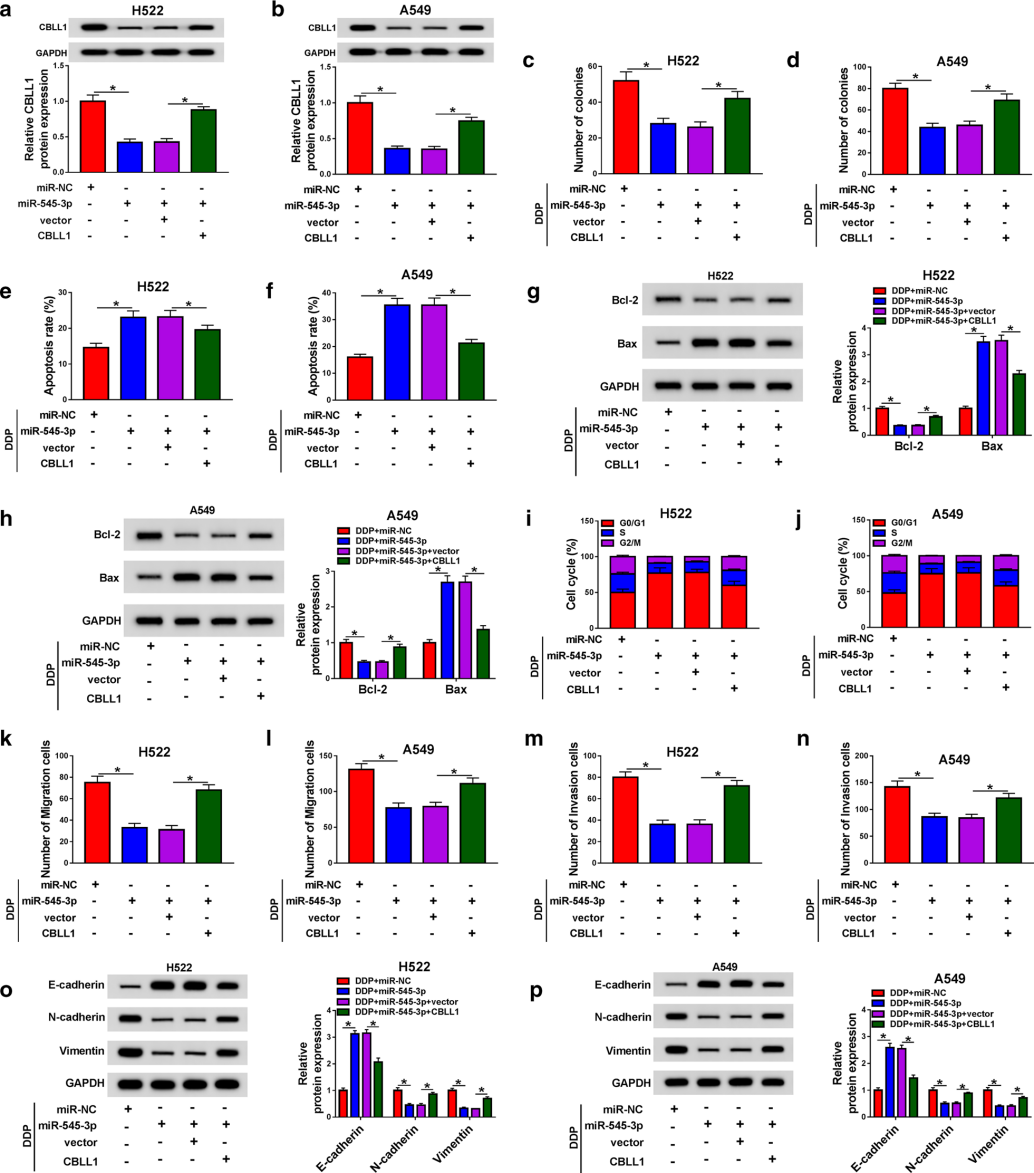
MiR-545-3p inhibits the malignant potential of NSCLC cells stimulated with DDP via inhibiting CBLL1. We co-transfected miR-NC, miR-545-3p, miR-545-3p + vector or miR-545-3p + CBLL1 into H522 and A549 cells. (**a, b**) The protein abundance of CBLL1 was measured in NSCLC cells by western blot assay. **c, d** The capacity of colony formation in NSCLC cells exposed to DDP was examined through colony formation assay. (**e, f**) The apoptosis of NSCLC cells stimulated with DDP was analyzed through flow cytometry. **g, h** Western blot assay was performed to measure the apoptosis-related proteins in NSCLC cells. **i, j** The cell cycle of NSCLC cells treated with DDP was analyzed using flow cytometry. **k–n** Transwell migration and invasion assays were applied to analyze the capacities of migration and invasion in NSCLC cells stimulated with DDP. **o, p** The protein levels of E-cadherin, N-cadherin and Vimentin were measured in NSCLC cells exposed to DDP by western blot assay. *P < 0.05

We wondered whether miR-545-3p played an anti-tumor role through targeting CBLL1 in NSCLC. CBLL1 overexpression recovered the abundance of CBLL1 in NSCLC cells, which was decreased by the transfection of miR-545-3p mimic (Fig. 6a, b). Functional experiments showed that the accumulation of miR-545-3p caused significant inhibition on the colony formation, cell cycle and metastasis along with a marked promotion on the apoptosis of NSCLC cells treated with DDP (Fig. 6c–n), and the co-transfection of CBLL1 and miR-545-3p recovered the malignant potential of NSCLC cells. The changes in the expression of metastasis-related proteins (E-cadherin, N-cadherin and Vimentin) also suggested that miR-545-3p repressed the metastasis of DDP-triggered NSCLC cells through down-regulating CBLL1 (Fig. 6o, p). Moreover, miR-545-3p accumulation promoted the necrosis of NSCLC cells treated with DDP, and the addition of CBLL1 regained the viability of NSCLC cells (Additional file 1: Figure S1c).

### Circ_0072083 depletion further aggravates DDP-mediated suppression of NSCLC tumor growth in vivo

**Fig. 7:**
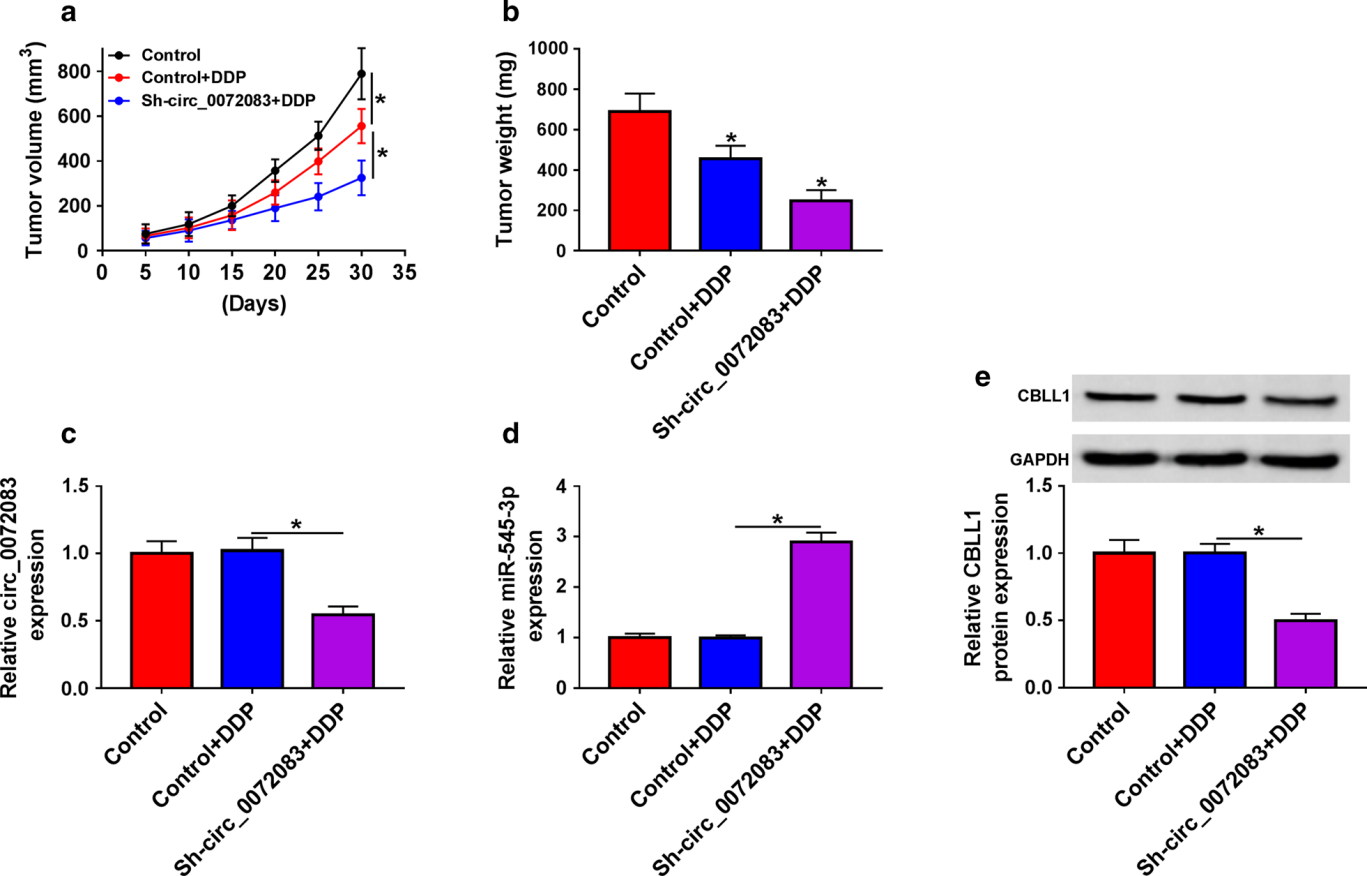
Circ_0072083 depletion further aggravates DDP-mediated suppression of NSCLC tumor growth in vivo. **a** The volume of tumors was measured after injection for 5, 10, 15, 20, 25 or 30 days. **b** The mice were killed after injection for 30 days, and the weight of tumors was measured. **c, d** The expression of circ_0072083 and miR-545-3p was detected in xenograft tumors by qRT-PCR. **e** Western blot was performed to determine the protein expression of CBLL1 in tumor tissues. *P < 0.05

We further tested the effects of circ_0072083 and DDP in xenograft tumor mice. The NSCLC tumors formed in DDP-treated group were lesser than those in the control group (Fig. 7a, b). Furthermore, the depletion of circ_0072083 in NSCLC tumors caused a further decrease in the size and weight of NSCLC tumors. The enrichment of circ_0072083, miR-545-3p and CBLL1 was measured in xenograft tumors. As mentioned in Fig. 7c–e, the abundance of circ_0072083 and CBLL1 was reduced in Sh-circ_0072083 + DDP group compared with that in Control + DDP group, while the expression of miR-545-3p revealed a reverse phenomenon.

## Discussion

CircRNAs have been reported to be associated with the progression of cancers [20, 21]. The abnormal expression of circRNAs was related to the development of NSCLC. For example, Wang et al. reported that circ_0067934 depletion blocked the growth and motility of NSCLC cells [22]. Li et al. proved that the high expression of circ_0016760 promoted the progression of NSCLC via miR-1287/GAGE1 signaling [23]. Liu et al. found that circ_0001649 repressed the progression of NSCLC through miR-331-3p and miR-338-5p [24]. This study showed that circ_0072083 was highly expressed in NSCLC tissues and cells. Besides, the silencing of circ_0072083 aggravated DDP-induced inhibition of colony formation, cell cycle and metastasis and DDP-mediated up-regulation of apoptosis and necrosis of NSCLC cells. The pro-cancer role of circ_0072083 in NSCLC was in alignment with the former article [12].

CircRNAs could bind to miRNAs and function as miRNAs sponges [20]. Accumulating articles have reported the downstream miRNAs of circ_0072083 in diverse cancers [25–27]. Herein, miR-545-3p was verified as a target of circ_0072083 in NSCLC cells. MiR-545-3p was an anti-cancer gene in multiple cancers. Chang et al. proved that miR-545-3p inhibited the proliferation, migration and invasion of hepatocellular carcinoma cells via MT1M [28]. Song et al. found that miR-545 hampered the growth of pancreatic ductal adenocarcinoma cells via RIG-1 [29]. In this study, we found that circ_0072083 promoted the progression of DDP-treated NSCLC cells via negatively regulating miR-545-3p.

Next, CBLL1 was predicted and confirmed as a target of miR-545-3p through Targetscan software and dual-luciferase reporter assay, respectively. CBLL1 was up-regulated in NSCLC and breast cancer [18, 19, 30]. Consistent with the previous studies, we found that the expression of CBLL1 was elevated in NSCLC tissues and cells. Besides, CBLL1 served as the downstream gene of circ_0072083/miR-545-3p to protect NSCLC cells against DDP-induced damage. The role of CBLL1 was also confirmed through establishing murine xenograft model in vivo.

## Conclusion

Collectively, circ_0072083 was found to be an oncogene in NSCLC, and circ_0072083 protected NSCLC cells against DDP-triggered injury through miR-545-3p/CBLL1 axis (Additional file 1: Figure S3).

## Supplementary information

**Additional file 1: Figure S1.** The influence of DDP, circ_0072083, miR-545-3p and CBLL1 on the necrosis of NSCLC cells. **a** The viability of NSCLC cells treated with si-NC, si-circ_0072083, DDP + si-NC or DDP + si-circ_0072083 was detected by LDH cytotoxicity assay kit. **b** LDH cytotoxicity assay kit was used to measure the necrosis of DDP-induced NSCLC cells transfected with si-NC, si-circ_0072083, si-circ_0072083 + anti-miR-NC or si-circ_0072083 + anti-miR-545-3p. **c** The viability of DDP-treated NSCLC cells transfected with miR-NC, miR-545-3p, miR-545-3p + vector or miR-545-3p + CBLL1 was determined through using LDH cytotoxicity assay kit. *P < 0.05.

**Additional file 2: Figure S2.** Circ_0072083 intervention has no significant effects on the colony formation and apoptosis of NSCLC cells. **a** The knockdown efficiency of si-circ_0072083 in BEAS-2B cells was examined by qRT-PCR. **b** Colony formation assay was employed to assess the colony formation ability of NSCLC cells transfected with si-NC or si-circ_0072083. **c** The apoptosis rate of NSCLC cells transfected with si-NC or si-circ_0072083 was evaluated by flow cytometry. *P < 0.05.

**Additional file 3: Figure S3.** Circ_0072083 interference enhances the inhibitory effect of cisplatin on the malignance of NSCLC cells via miR-545-3p/CBLL1 axis.

## Data Availability

The data that support the findings of this study are available on request from the corresponding author.

## Abbreviations

NSCLC: Non-small-cell lung cancer
qRT-PCR: Quantitative real time polymerase chain reaction
ZFR: Zinc finger RNA binding protein
CBLL1: Cbl proto-oncogene like 1
RIP: RNA immunoprecipitation
