# Supplementary figures and images for: Circ_0072083 interference enhances growth-inhibiting effects of cisplatin in non-small-cell lung cancer cells via miR-545-3p/CBLL1 axis
Source: Cancer Cell Int. 2020 Mar 12;20:78. doi: 10.1186/s12935-020-1162-x (PMC7066755; doi:10.1186/s12935-020-1162-x)

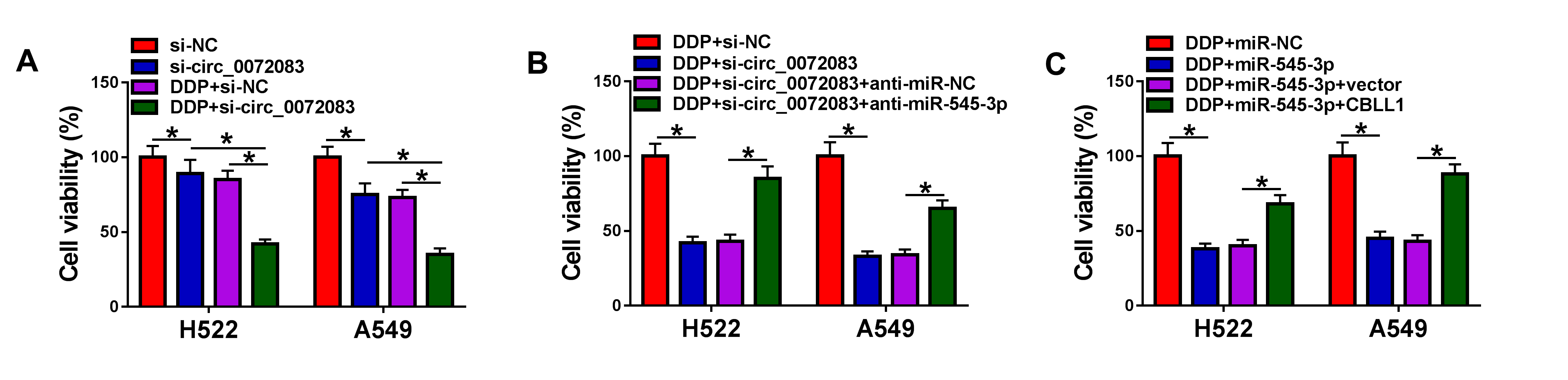

Supplement: Supplementary file 1 — Additional file 1: Figure S1. The influence of DDP, circ_0072083, miR-545-3p and CBLL1 on the necrosis of NSCLC cells. a The viability of NSCLC cells treated with si-NC, si-circ_0072083, DDP + si-NC or DDP + si-circ_0072083 was detected by LDH cytotoxicity assay kit. b LDH cytotoxicity assay kit was used to measure the necrosis of DDP-induced NSCLC cells transfected with si-NC, si-circ_0072083, si-circ_0072083 + anti-miR-NC or si-circ_0072083 + anti-miR-545-3p. c The viability of DDP-treated NSCLC cells transfected with miR-NC, miR-545-3p, miR-545-3p + vector or miR-545-3p + CBLL1 was determined through using LDH cytotoxicity assay kit. *P < 0.05. [file 12935_2020_1162_MOESM1_ESM.tif]

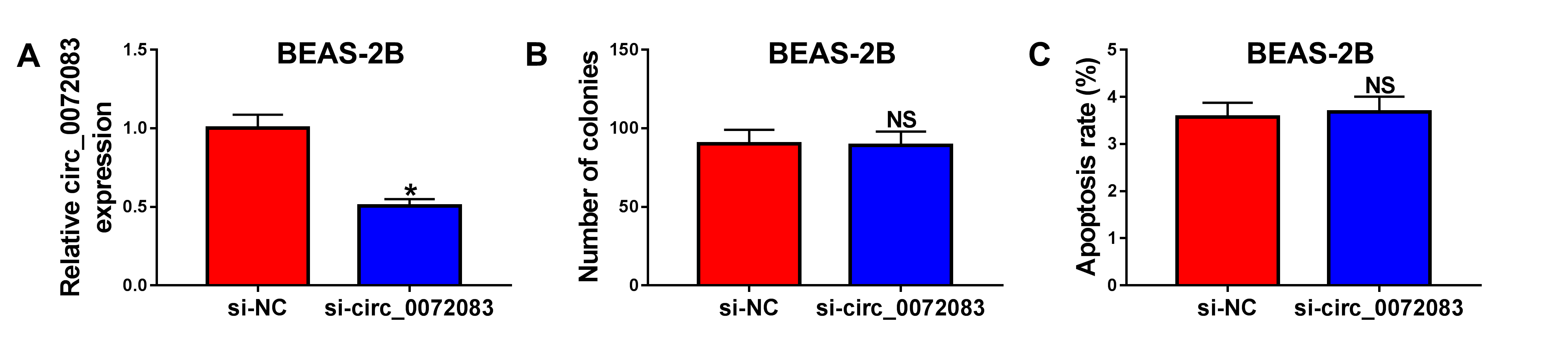

Supplement: Supplementary file 2 — Additional file 2: Figure S2. Circ_0072083 intervention has no significant effects on the colony formation and apoptosis of NSCLC cells. a The knockdown efficiency of si-circ_0072083 in BEAS-2B cells was examined by qRT-PCR. b Colony formation assay was employed to assess the colony formation ability of NSCLC cells transfected with si-NC or si-circ_0072083. c The apoptosis rate of NSCLC cells transfected with si-NC or si-circ_0072083 was evaluated by flow cytometry. *P < 0.05. [file 12935_2020_1162_MOESM2_ESM.tif]

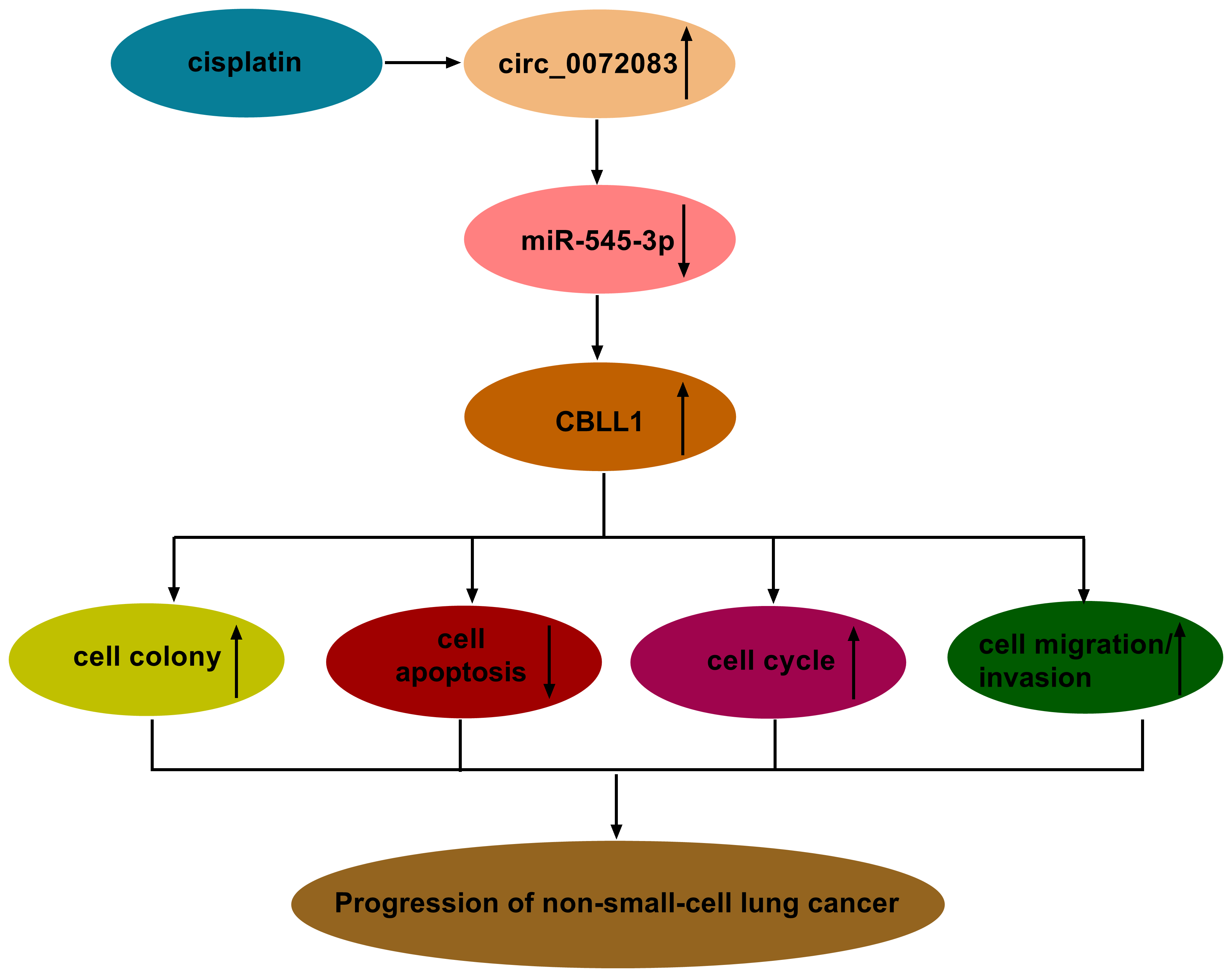

Supplement: Supplementary file 3 — Additional file 3: Figure S3. Circ_0072083 interference enhances the inhibitory effect of cisplatin on the malignance of NSCLC cells via miR-545-3p/CBLL1 axis. [file 12935_2020_1162_MOESM3_ESM.tif]
